# Editorial: Novel adjuvant systems that generate pathogen specific T cell responses following immunization for infectious disease vaccine formulations

**DOI:** 10.3389/fimmu.2024.1467461

**Published:** 2024-08-14

**Authors:** Amanda M. Burkhardt, Szu-Wen Wang, Ali M. Harandi

**Affiliations:** ^1^ Alfred E. Mann School of Pharmacy and Pharmaceutical Sciences, Department of Clinical Pharmacy, University of Southern California, Los Angeles, CA, United States; ^2^ Department of Chemical & Biomolecular Engineering, University of California, Irvine, Irvine, CA, United States; ^3^ Department of Microbiology and Immunology, Institute of Biomedicine, Sahlgrenska Academy, University of Gothenburg, Gothenburg, Sweden

**Keywords:** adjuvant, T cell stimulating adjuvants, cellular immune response, vaccine, vaccines for infectious disease

Adjuvants are an essential component of subunit vaccine formulations and are gaining wider experimental use in attenuated and nucleic acid vaccine formulations. Adjuvants increase the immunogenic response to the immunizing antigens or agent and can influence the type of immune response to vaccination. First generation adjuvants, including alum and squalene oil-in-water emulsions, function as antigen delivery systems by creating depots of trapped antigen at the injection site. This class of adjuvants is widely available for vaccine formulations and have been approved for use in many licensed vaccines. Second generation adjuvants are ligands for pathogen-associated molecular pattern (PAMP) recognition receptors, such as Toll-like receptors (TLRs), that activate antigen presenting cells through their cognate receptor. Although the FDA has begun approving new adjuvants beyond first generation systems, such as TLR agonists, there remains a paucity of T cell stimulating adjuvanting agents available for use in human vaccine formulations. As a result, there is still a lack of effective vaccine candidates for many pathogens that require a cellular immune response for protection.

Despite the robust and protective immune responses elicited by whole-organism vaccines, this technology is not relevant for every infectious pathogen requiring a vaccine. For some pathogens the risk of reactivation makes them too dangerous to be given as a whole organism vaccine formation, and others can be easily weaponized as bioterrorism agents and therefore cannot be realistically scaled up for the purpose of immunization. Still, other whole-organism vaccine formulations elicit far too robust immune responses to be safe. Subunit vaccines offer safe solutions to each of these issues. However, subunit vaccines require the inclusion of an adjuvant to generate an immunogenic response to immunization. The status quo as it pertains to adjuvant formulation in subunit vaccine development is to use adjuvanting agents that preferentially skew towards humoral responses and away from cellular responses to the immunizing antigens. For some pathogens, robust humoral responses to immunization are sufficient to provide protection upon exposure with the pathogenic organism. However, many pathogens, including bacterial species like *Coxiella burnetii* and *Mycobacterium tuberculosis*, require effective cellular responses for protection against challenge. Interestingly, many of these pathogens, including several neglected tropic diseases and bioterrorism agents, are still lacking effective vaccines, underscoring the need for a better knowledge of adjuvants that generate T cell responses, and an expanded understanding of the mechanisms through which adjuvants that stimulate cellular immune responses confer protection.

Several significant innovations and advances have been made in the field of T cell stimulating adjuvant systems, including STING agonists and nanoparticles, yet these systems are not widely used in approved vaccine formulations, despite the immediate impact these adjuvants could have on human health and disease prevention.

The review article in this Research Topic presents a comprehensive overview of current vaccines and adjuvant technologies that specifically target T cell responses while also discussing potential receptor and cellular targets that could further increase cellular responses to vaccination (Ung et al.). This review is an excellent overview of the current state of this field and a critical primer for those interested in learning more about this field of T cell stimulating adjuvants and the potential they hold for preventing infectious diseases.

The three research articles in this Research Topic detail the impacts these novel T cell stimulating adjuvants can have in three different infectious diseases: *Yersinia pestis* (Galloway et al.), influenza virus (Jangra et al.), and porcine epidemic diarrhea virus (PEDV) (Wang et al.). Two of these have significant relevance for human health (*Y. pestis* and influenza virus) while the third can make significant impressions in the agricultural space by controlling an important swine coronavirus that has been recently introduced into North American herds. These articles demonstrate that these novel T cell stimulating adjuvants have the potential to impact issues beyond direct human health by preventing agricultural outbreaks and pandemics, which are becoming more prevalent and raise significant economic and global food access concerns.

Although historically these adjuvant platforms have not been the first selection for vaccine formulations, the articles in this Research Topic clearly illustrate their ability to provide protection against a wide range of infectious diseases that effect humans and other species. It is with both excitement and hope that the field will continue to watch this specific class of adjuvants continue to be developed and used in vaccines to provide protection against both old and emerging infectious diseases.

